# Supplementary material for: Modeling glioblastoma heterogeneity as a dynamic network of cell states
Source: Mol Syst Biol. 2021 Sep 16;17(9):e10105. doi: 10.15252/msb.202010105 (PMC8444284; doi:10.15252/msb.202010105)
Supplement: Supplementary file 5 — Source Data for Figure 3 [file MSB-17-e10105-s001.zip › Figure3A_sourcedata/GSEA_3065/nosigdb_state3.GseaPreranked.1622718426028/ALLEN_114_OPCL1_6MYT1.html]

Details for gene set ALLEN\_114\_OPCL1\_6MYT1[GSEA]

|  || Dataset | state3 |
| Phenotype | NoPhenotypeAvailable |
| Upregulated in class | na\_pos |
| GeneSet | ALLEN\_114\_OPCL1\_6MYT1 |
| Enrichment Score (ES) | 0.82560205 |
| Normalized Enrichment Score (NES) | 2.180435 |
| Nominal p-value | 0.0 |
| FDR q-value | 1.7678573E-4 |
| FWER p-Value | 0.001 |
Table: GSEA Results Summary

  

Fig 1: Enrichment plot: ALLEN\_114\_OPCL1\_6MYT1      
 Profile of the Running ES Score & Positions of GeneSet Members on the Rank Ordered List

  

| PROBE | GENE SYMBOL | GENE\_TITLE | RANK IN GENE LIST | RANK METRIC SCORE | RUNNING ES | CORE ENRICHMENT || 1 | PTPRZ1 |  |  | 14 | 0.532 | 0.1550 | Yes |
| 2 | PTN |  |  | 22 | 0.493 | 0.2994 | Yes |
| 3 | SOX6 |  |  | 88 | 0.358 | 0.3974 | Yes |
| 4 | PDGFRA |  |  | 115 | 0.326 | 0.4904 | Yes |
| 5 | SEMA5A |  |  | 121 | 0.324 | 0.5850 | Yes |
| 6 | VCAN |  |  | 293 | 0.245 | 0.6376 | Yes |
| 7 | DOCK10 |  |  | 395 | 0.219 | 0.6906 | Yes |
| 8 | PLEKHH2 |  |  | 440 | 0.211 | 0.7477 | Yes |
| 9 | OLIG1 |  |  | 602 | 0.186 | 0.7839 | Yes |
| 10 | COL11A1 |  |  | 684 | 0.173 | 0.8256 | Yes |
| 11 | APOD |  |  | 1113 | 0.129 | 0.8145 | No |
| 12 | MAML2 |  |  | 1883 | 0.082 | 0.7505 | No |
| 13 | MEGF11 |  |  | 2022 | 0.075 | 0.7568 | No |
| 14 | NXPH1 |  |  | 3773 | 0.021 | 0.5626 | No |
| 15 | NCKAP5 |  |  | 3860 | 0.018 | 0.5582 | No |
| 16 | NKAIN3 |  |  | 4346 | 0.005 | 0.5042 | No |
Table: GSEA details [plain text format]

  

Fig 2: ALLEN\_114\_OPCL1\_6MYT1: Random ES distribution      
 Gene set null distribution of ES for **ALLEN\_114\_OPCL1\_6MYT1**
